# Prediction of response to anti-EGFR antibodies in metastatic colorectal cancer: looking beyond EGFR inhibition

**DOI:** 10.3389/fimmu.2012.00409

**Published:** 2013-01-07

**Authors:** Alessandro Ottaiano, Maurizio Capuozzo, Guglielmo Nasti, Piera Maiolino, Valentina De Angelis, Stefania Scala, Rosario V. Iaffaioli

**Affiliations:** ^1^Department of Colorectal Oncology at the National Cancer Institute, “G. Pascale” foundation, via M. SemmolaNaples, Italy; ^2^Department of Pharmacy at the ASL-Naples-3, via Marittima 3/BErcolano, NA, Italy; ^3^Department of Pharmacy at the National Cancer Institute, “G. Pascale” foundation, via M. SemmolaNaples, Italy; ^4^Institute of Psychological and Systemic Medicine, via F. Giordani 30Naples, Italy; ^5^Department of Cancer Immunology at the National Cancer Institute, “G. Pascale” foundation, via M. SemmolaNaples, Italy

**A commentary on**

**The evolving role of monoclonal antibodies in colorectal cancer: early presumptions and impact on clinical trial development**

by Eng, C. (2010). Oncologist 15, 73–84.

One of the most successfully approach in the treatment of metastatic colorectal cancer (mCRC) is the inhibition of the Epidermal Growth Factor Receptor (EGFR) pathway by antibodies (cetuximab and panitumumab). Notably, randomized trials with anti-EGFR antibodies have shown a significant impact of KRAS [wild type (wt) vs. mutated (mut)] on response and prognosis: the presence of KRAS activating mutations was found to be associated with reduced biological and clinical activity for the treatment (response rate in mut <20% vs. wt >50%). Thus, the mutational status of KRAS is now a widely accepted criteria for selection of patients who would be most likely to respond to these treatments. In the next future, other genes involved in the EGFR pathway could have a role in the prediction of treatment response (BRAF, PIK3CA, PTEN, etc.) (De Roock et al., 2011).

Cetuximab is an IgG1 monoclonal antibody, it binds specifically to the extracellular domain of EGFR inhibiting downstream proliferative, anti-apoptotic and neoangiogenetic signals in kras wt tumors and it has clinical efficacy in mCRC (Eng, 2010). However, one of the accepted anti-tumor mechanism is the antibody-dependent cell-mediated cytotoxicity (ADCC) in which Fc region of the antibody binds to the FcγRs (Fragment c Gamma Receptors) expressed by immune effector cells (Natural Killer cells, macrophages, etc.) (Kohrt et al., 2012). However, the scenario is very complex and the result is not the simple sum of the above phenomena. Very recently, it has been demonstrated that immunologic mechanisms can cooperate (ADCC) but also antagonize with the inhibition of EGFR/kras signal. In fact, CD163+ “tumor-promoting” M2 macrophages which can be abundant in the microenvironment of colorectal carcinomas are activated by cetuximab and in turn they release anti-inflammatory and tumor-promoting mediators, including IL-10 and VEGF (Pander et al., 2011). Furthermore, both ADCC and cetuximab-induced macrophage responses can be more pronounced for FcγRIIIa 158-Val (high-affinity receptor for Fc) carriers (Tsuchiya et al., 2007; Pander et al., 2011). The different abundance and activity of CD163 + M2 macrophages in tumor environment could explain the contrasting results reported in literature on the role of FcγR polymorphisms in mCRC (Zhang et al., 2007; Bibeau et al., 2009).

Very recently, we have demonstrated that homozygous carriers of the 158V allele of the FcγRIIIa show a high response rate and a significantly improved prognosis in kras wt mCRC (Calemma et al., 2012). This was consistent with the hypothesis that variants of human IgG-receptors can influence the extent of ADCC and, thus, response to anti-EGFR therapy. We made, however, the intriguing observation that FcγRIIIa polymorphisms had a prognostic power also in the entire series, including patients with mut kras who did not receive anti-EGFR therapy (data not shown). To confirm this observation, we are extending the analysis of FcγRIIIa polymorphisms to all mCRC patients referring to our center. Our speculation is that ADCC could be triggered by “endogenous” anti-tumor antibodies binding to “high-affinity” Fcγ R and might be capable of mediating a clinically relevant anti-tumor activity. Such antibodies could be present and work also in mutant kras mCRC patients. The hypothesis that “endogenous” rather than “therapeutic” antibodies might trigger such activity is fascinating but difficult to demonstrate and could be responsible of long-term clinical stabilizations after surgery and/or radio and/or chemotherapy that we see in clinical practice. Indirectly, increased rates of antibody-mediated autoimmune diseases in 158V carriers suggest that the polymorphism also plays a relevant role in the binding of endogenous antibodies (Matsumoto et al., 2005).

ADCC could be also responsible of responses to anti-EGFR antibodies seen in KRAS mut tumors. In fact, Ashraf et al. (2012) have demonstrated that higher EGFR expression can predict susceptibility to cetuximab-induced immune killing of CRC cells occurring independently of KRAS/BRAF/PIK3CA mutations (in press on *Proc. Natl. Acad. Sci. U.S.A.*). Therefore, administration of anti-EGFR antibodies may be considered in CRC tumors with higher target expression and favorable FcγR polymorphisms. However, the context is very complex and other factors can influence the response to anti-tumor antibodies: previous and/or concomitant therapies, HLA expression, cytokines production, immune cell receptors repertoire, etc.

Study of interactions between host and tumors should be urgently improved to optimize the prediction of response to therapeutic antibodies in mCRC.
